# More than Just an Immunosuppressant: The Emerging Role of FTY720 as a Novel Inducer of ROS and Apoptosis

**DOI:** 10.1155/2018/4397159

**Published:** 2018-03-28

**Authors:** Teruaki Takasaki, Kanako Hagihara, Ryosuke Satoh, Reiko Sugiura

**Affiliations:** Laboratory of Molecular Pharmacogenomics, Department of Pharmaceutical Sciences, Faculty of Pharmacy, Kindai University, 3-4-1 Kowakae, Higashi-Osaka, 577-8502 Osaka, Japan

## Abstract

Fingolimod hydrochloride (FTY720) is a first-in-class of sphingosine-1-phosphate (S1P) receptor modulator approved to treat multiple sclerosis by its phosphorylated form (FTY720-P). Recently, a novel role of FTY720 as a potential anticancer drug has emerged. One of the anticancer mechanisms of FTY720 involves the induction of reactive oxygen species (ROS) and subsequent apoptosis, which is largely independent of its property as an S1P modulator. ROS have been considered as a double-edged sword in tumor initiation/progression. Intriguingly, prooxidant therapies have attracted much attention due to its efficacy in cancer treatment. These strategies include diverse chemotherapeutic agents and molecular targeted drugs such as sulfasalazine which inhibits the CD44v-xCT (cystine transporter) axis. In this review, we introduce our recent discoveries using a chemical genomics approach to uncover a signaling network relevant to FTY720-mediated ROS signaling and apoptosis, thereby proposing new potential targets for combination therapy as a means to enhance the antitumor efficacy of FTY720 as a ROS generator. We extend our knowledge by summarizing various measures targeting the vulnerability of cancer cells' defense mechanisms against oxidative stress. Future directions that may lead to the best use of FTY720 and ROS-targeted strategies as a promising cancer treatment are also discussed.

## 1. Introduction

**Figure 1:**
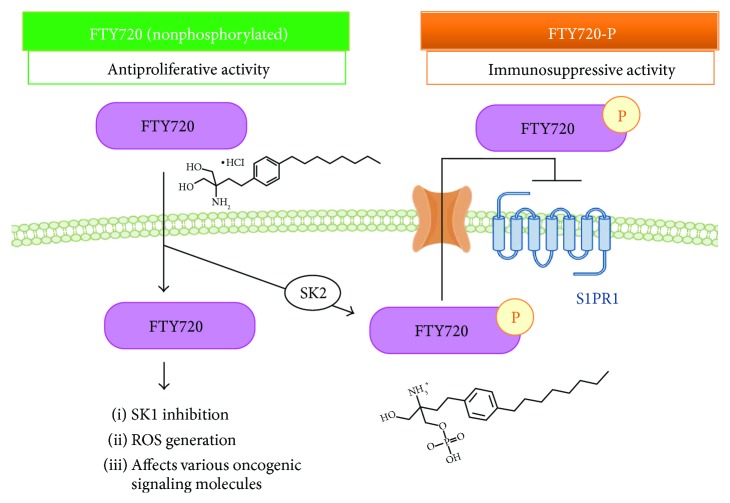
FTY720 has anticancer properties as well as immunosuppressive activity. The nonphosphorylated form of FTY720 exhibits antiproliferative activity through inhibition of SK1 and generation of ROS and affects oncogenic signaling molecules and so on. In contrast, phospho-FTY720 (FTY720-P), which is converted from FTY720 by sphingosine kinases such as SK2, is exported through transporter and acts as a functional antagonist at S1P receptor thereby inhibiting lymphocyte egress from lymphoid organs. Chemical structures of FTY720 and FTY720-P are shown.

FTY720 (also known as Fingolimod/Gilenya) is a potent immunosuppressant which was approved as a first-line therapy for relapsing forms of multiple sclerosis in 2010 [1]. FTY720 exerts its immunosuppressive effects as a first-in-class S1P (sphingosine-1-phosphate) receptor modulator [2]. FTY720 is a structural analogue of sphingosine derived from myriocin (ISP-1), a metabolite of the fungus *Isaria sinclairii*. FTY720 is phosphorylated by sphingosine kinases (SKs) in the cell (most importantly SK2) [3]. In this aspect, FTY720 is a prodrug, with its immunosuppressive effects elicited following its phosphorylation by SKs (to form phospho-FTY720 or FTY720-P). Phospho-FTY720 subsequently causes the internalization of S1P receptors, which results in lymphopenia by lymphocyte sequestration in lymphoid tissues, thus preventing them from moving to the central nervous system thereby causing a relapse in multiple sclerosis (Figure 1) [2].

Notably, however, in addition to its well-known mode of action as an S1P receptor modulator with strong immunosuppressive properties, it has now become clear that FTY720 possesses a multitude of other effects on cells. Especially, ample evidence suggests that FTY720 in its unphosphorylated form has anticancer and antimetastatic properties (Figure 1) [4]. The anticancer activity of FTY720 has been reported in various cancer cell types including breast cancers [5–7], bladder cancer [8], glioblastoma [5, 9, 10], hepatocellular carcinoma [11–13], leukemia, and malignant mesothelioma [14, 15], implying that FTY720 action is involved in multiple intracellular signaling pathways related to cancer signaling.

There are some potential mechanisms responsible for the antiproliferative properties mediated by FTY720. One of the well-studied mechanisms involves the inhibition of sphingosine kinase 1 (SK1) by FTY720 [16]. SK1 is a protooncogene and is considered a potential target for cancer therapy, based on the rationale that SK1 activation contributes to cancer progression, ranging from migration and proliferation to angiogenesis/lymphangiogenesis [17–19]. FTY720 is also known to downregulate prosurvival mitogen-activated protein kinase (MAPK) and phosphatidylinositol 3-kinase/Akt pathways and upregulate stress-activated kinases (SAPKs) such as p38 [14, 20].

Furthermore, the importance of FTY720-mediated ROS generation in inducing apoptosis, a popular target of many cancer treatment strategies, has been highlighted as an additional potential mechanism of FTY720-dependent cancer cell death (Figure 1) [9, 11, 12, 21–23]. Intriguingly, these antiproliferative effects of FTY720 are mediated independent of S1P receptors and thus appear facilitated by nonphosphorylated FTY720 via modulation of a range of other targets [24]. Although various signaling molecules such as protein phosphatase 2A, JNK, Akt, MAPK, Rho-GTPase, and sphingosine kinase have been suggested to mediate anticancer effects associated with FTY720 [7, 9, 15, 23], signaling networks orchestrated by FTY720 as well as the mechanisms as to how FTY720 induces ROS and cell death remain elusive.

Recent advances in chemical genomics studies have considerably contributed to elucidating the mechanisms of drug action as well as various gene networks relevant to susceptibility to side effects and/or efficacy of various drugs. Especially, the fission yeast *Schizosaccharomyces pombe* (*S. pombe*) is a powerful model system for studying the mechanism(s) of drug action and genetic contexts associated with drug sensitivity and/or resistance, as evidenced by the demonstration of calcineurin and a phosphatidylinositol 3-kinase homologue as the target of the FK506-FKBP complex and of the rapamycin-FKBP complex, respectively [25–28]. Importantly, FTY720, but not FTY720-P (phosphorylated FTY720), elicited cell death effects by inducing ROS and subsequent activation of the highly conserved stress-activated MAPK (SAPK) signaling in fission yeast [29]. Intriguingly, the fission yeast genome does not express the S1P receptor, which, in combination with its powerful genetic tools and resources, would be beneficial to clearly delineate the components and signaling pathways relevant to FTY720-mediated ROS induction and cell death. Importantly, the fission yeast chemical genomic screen also unraveled as-yet-identified targets for combination therapy by enhancing the effect of FTY720 on ROS-mediated signaling.

The ROS-modulating anticancer strategy is an important and long-standing question, and a source of much controversy, partly due to the double-faced nature of ROS in the control of cell proliferation and cell death [30]. Notably, however, mounting evidence highlights the efficacy of targeting the vulnerability of cancer cells to ROS defense mechanisms. In this review, we introduce the role of FTY720 as a ROS inducer and its relevance to cancer therapy.

## 2. Apoptotic Effects of FTY720 Involving ROS

FTY720 has been shown to induce apoptosis in various human cancer cell lines, including multiple myeloma cells [31], liver [32, 33], prostate [14], breast [6, 7, 34], kidney [35], and bladder cancer cells [8]. Notably, the antitumor property of FTY720 is largely mediated via the involvement of S1P receptor-independent mechanisms [7, 11, 18, 34, 36, 37]. For example, Yoshino et al. reported that phosphorylated FTY720 (FTY720-P) and SEW2871, S1P1-selective agonists, did not induce apoptosis of the human microglia cell line HMO6 and suggest that FTY720-non-P-induced apoptosis of HMO6 is independent of S1P receptor binding [37]. Furthermore, Neviani et al. showed that FTY720 but not its immunosuppressive phosphorylated form FTY720-P exerts antileukemic activity and suggested that FTY720 represents a powerful therapeutic tool as it has the potential to treat Ph(+) and Ph(−) myeloproliferative disorders [38].

Important findings regarding the functional connection between FTY720-mediated apoptosis and ROS have been proposed by Chen's group who initially investigated the mechanisms by which FTY720 induces apoptosis in hepatocellular carcinoma cells (HCC) [12]. They demonstrated that FTY720 regulates antitumor effects via activating NADPH oxidase by upregulating the gp91^phox^ subunit expression and subsequently activating PKC*δ*-caspase-3 signaling in HCC. They further developed these findings by exploiting OSU-2S, which represents a nonphosphorylated form of FTY720. In contrast to FTY720, OSU-2S was not phosphorylated by sphingosine kinase 2 (SK2) *in vitro* and did not cause S1P1 receptor internalization in HCC cells or T lymphocyte homing in immunocompetent mice. This group successfully demonstrated the efficacy of the nonimmunosuppressive FTY720 analogue as an antitumor agent, which is evidenced by the fact that OSU-2S exhibited higher potency than FTY720 in suppressing HCC cell growth through PKC*δ* activation [11]. These findings highlighted that OSU-2S, devoid of S1P1 receptor modulating activity, is a novel PKC*δ*-targeted antitumor agent via ROS generation and has clinical value in therapeutic strategies for HCC.

In addition, accumulating evidence suggests that FTY720-induced cytotoxicity occurred dependent on the generation of ROS in various cancer cells. These include human glioblastoma cells, mantle cell lymphoma, and myeloma cells [9, 23, 39]. Intriguingly, several papers suggested the involvement of ROS-induced autophagy as a mechanism in FTY720-mediated tumor-suppressive effects including glioblastoma cells and ovarian cancer cells [9, 40]. Bai et al. reported that FTY720 induces autophagy-associated apoptosis in human oral squamous carcinoma cells as evidenced by LC3B-II conversion, reduced p62 expression, and autophagosome accumulation through ROS production [41]. Importantly, inhibition of autophagy attenuates FTY720-induced cytotoxicity, and FTY720-mediated cytotoxicity is dependent on the generation of ROS, thus suggesting an intricate interplay between autophagy and apoptosis in mediating the tumor-suppressive effect of FTY720. In addition, Zhang et al. reported that FTY720 induced extrinsic apoptosis, necroptosis, and autophagy in human glioblastoma cells both *in vivo* and *in vitro* and this induction is dependent on FTY720-mediated activation of the ROS-JNK-p53 signaling pathway, which resulted in suppressing AKT phosphorylation [9]. The authors demonstrated that FTY720-P showed no significant cytotoxic effects, again confirming the involvement of phosphorylation-independent and S1P signaling-independent mechanisms involving the cytotoxic effect of FTY720 (Figure 1).

Because growing evidence has suggested the involvement of ROS in the modulation of various autophagy-regulating proteins such as ATG4, AMPK, and NF-*κ*B and in cancer inhibition [42], a deeper investigation is required regarding the functional connection between these signaling proteins and FTY720 treatment.

## 3. A Chemical Genetic Screen Revealed a Network Orchestrated by FTY720-Mediated ROS Homeostasis

Chemical genetics is the study of genes through small-molecule perturbation. Chemical genetic screening is a phenotypic screening methodology that systematically tests the efficacy of small molecules. Recent advancements in chemical genetics and chemical genomics have opened new avenues for development of clinically relevant drug treatments [43]. Systematic mapping of genetic networks by high-throughput chemical genetics screens has given extensive insights in connections between genetic pathways. The budding yeast *Saccharomyces cerevisiae* and the fission yeast *Schizosaccharomyces pombe* (*S. pombe*) are widely used model organisms, and yeast genetic methods are powerful tools for discovery of novel functions of genes.

Sugiura and coauthors performed a series of extensive studies using fission yeast chemical genetics/genomics approaches and elucidated genes to determine the sensitivity/tolerance to FK506, valproic acid, micafungin, and rapamycin [25–27, 44–52]. For example, Sugiura's group systematically isolated mutants synthetically lethal with calcineurin deletion by performing genome-wide approaches utilizing the calcineurin inhibitor FK506, which successfully revealed the calcineurin signaling network and demonstrated the functional interaction between calcineurin and genes involved in the Golgi/endosomal membrane traffic events [44]. In addition, they also established molecular genetic approaches to screen for genes and compounds that target the MAPK signaling pathway, which resulted in the identification of numerous regulators of MAPK signaling and compounds, such as ACA-28, with antiproliferative properties via MAPK signaling modulation [53]. Therefore, chemical genetic screening is an efficient way to discover and validate new druggable targets and identify potentially efficacious therapeutics.

**Figure 2:**
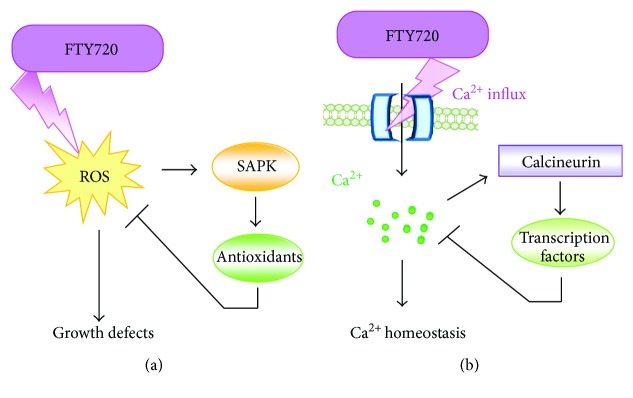
Cell death mechanisms mediated by FTY720 in fission yeast. (a) FTY720 stimulates production of ROS, which causes growth defects. Cytotoxicity of FTY720 is more pronounced in the cells that lack the components of the SAPK signaling pathway. (b) FTY720 stimulates Ca^2+^ influx, thereby stimulating calcineurin signaling. Cells lacking the components of the Ca^2+^/calcineurin signaling pathway are hypersensitive to FTY720.

Sugiura's group also embarked on the pharmacological exploitation of FTY720 to analyze the signaling pathways relevant to FTY720-induced cell death using fission yeast. Hagihara et al. showed that FTY720, but not FTY720-P, induced cytotoxicity [54]. They further investigated the underlying cell death mechanisms mediated by FTY720 and demonstrated that unphosphorylated FTY720, but not FTY720-P, stimulated ROS production and Ca^2+^ influx, followed by subsequent activation of the highly conserved Sty1/Spc1 stress-activated MAPK (SAPK) signaling and Ca^2+^/calcineurin signaling pathways, respectively (Figure 2) [29, 54].

The physiological importance of SAPK signaling and the Ca^2+^/calcineurin signaling pathways in the mechanisms of action as well as determinants of sensitivity/tolerance of FTY720 was further confirmed that deletion of the components of the two signaling pathways induced markedly higher sensitivities to FTY720. Especially, cells lacking the components of the SAPK pathway exhibited growth inhibition in the media containing FTY720 with enhanced ROS accumulation (Figure 2). Because SAPK signaling plays a central role in the defense mechanism against ROS/oxidative stress, the loss-of-function of SAPK signaling in combination with FTY720 treatment induced a higher ROS accumulation, thus indicating that an appropriate balance of ROS signaling plays a key role as a determinant of FTY720 cytotoxicity in fission yeast. Given the finding that the fission yeast genome does not express the S1P receptor homologue, together with the highly conserved nature of ROS production/regulation mechanisms as well as tremendous genetic resources/tools available, the above observations strongly demonstrated that this model organism has advantages and benefits in analyzing FTY 720 action in relevance to ROS and the cell death mechanism independent of its role as an S1P modulator.

## 4. Rationale for ROS-Inducing Agents as a Novel Therapeutic Measure in Cancer Therapy

As described above, several studies strongly suggested that FTY720 exhibits antitumor effects by increasing ROS generation in various cancer cells, and this inhibitory effect to tumor growth could be partially rescued by a ROS scavenger NAC (N-acetylcysteine). So, what is the rationale for inducing ROS to kill cancer cells? Accumulating evidence demonstrates the unique redox situation in tumor cells and suggests the use of ROS generation as a novel strategy for anticancer therapy as described below.

**Figure 3:**
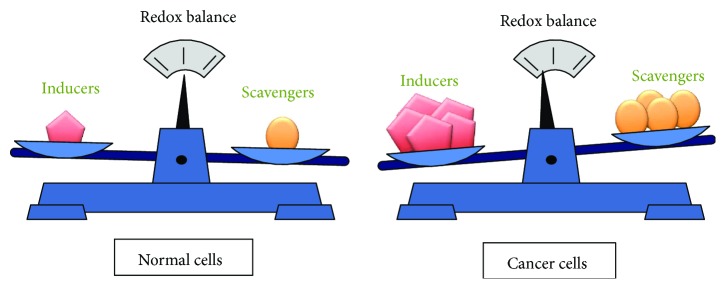
ROS levels are determined by a balance between ROS inducers and ROS scavengers. Determination of cellular redox status is achieved by a balance between ROS inducers (prooxidants) and ROS scavengers (antioxidants). Under physiological conditions, normal cells maintain redox balance with a low level of ROS inducers and ROS scavengers. In cancer cells, oncogenic signaling activation and/or metabolic alterations induce ROS generation, which also induce ROS scavengers to adapt oxidative stresses.

Reactive oxygen species (ROS) play an essential role in maintaining cellular homeostasis, and cells control ROS levels and redox status by the delicate balance between ROS generation with their elimination by ROS-detoxifying (scavenging) systems. Thus, tight regulation of both ROS inducer signaling and ROS scavenger signaling is thus required (Figure 3). ROS inducers include hypoxia, lack of exercise, smoking, air pollution, ER stress, radiation, and oncogene activation [55–58]. ROS scavengers include redox enzymes (including superoxide dismutase, glutathione peroxidase, and catalase), antioxidants (glutathione, thioredoxin, and peroxiredoxin), and dietary antioxidant compounds (vitamins C and E, polyphenols), as well as various transcription factors including NRF2, FOXO, and p53, that are involved in gene expression of enzymes with antioxidant functions [59–63].

**Figure 4:**
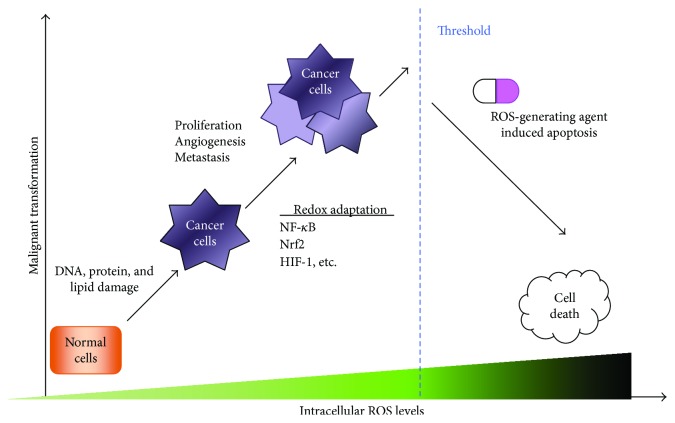
ROS paradox and the concept of prooxidant cancer therapy. In precancerous conditions, ROS levels are slightly elevated which facilitate characteristic carcinogenic and mutagenic processes, including DNA, protein, and lipid damages, and stimulate tumor cell proliferation. Persistent exposure to ROS induces redox adaptation, including activation of redox-sensitive transcription factors (e.g., NF-*κ*B, Nrf2, and HIF-1) that increase the expression of ROS-scavenging enzymes. Malignant cells exhibit higher steady-state levels of ROS due to an adaptive increase of antioxidant capacity. The high ROS levels in cancer cells render them more susceptible/vulnerable to further oxidative stress induced by exogenous ROS-generating agents. When the levels of ROS elevate above the threshold that cancer cells can adapt, cells can no longer survive leading to cell death.

Most cancer cells exhibit increased levels of ROS as compared with the normal counterparts, which is counteracted by an increased antioxidant capacity (Figure 3). A moderate increase in ROS is beneficial in promoting cell proliferation and differentiation, whereas excess levels of ROS that overwhelm the cellular antioxidant capacity are detrimental to cells, by causing oxidative damage to DNA, lipids, and proteins, which can contribute to development of tumors (Figure 4) [64]. Thus, an increase of ROS is considered to promote tumor initiation and progression as well as the maintenance of tumor cell phenotypes, by inducing prooncogenic signaling pathways, thereby serving as a rationale for antioxidant cancer therapy. Indeed, dietary antioxidants such as red wine and green tea polyphenols have long been recommended for cancer prevention. Notably, however, the use of several antioxidants, such as vitamin E or vitamin C, in cancer prevention is still controversial and complex and needs to be carefully evaluated [65]. For example, some data suggest antioxidants can ameliorate toxic side effects of cancer therapy without affecting treatment efficacy, whereas others suggest antioxidants interfere with chemotherapy which are largely dependent on ROS generation to induce cytotoxicity in tumors [66]. These contradictory effects of ROS and/or antioxidants have important implications for potential anticancer strategies that aim to modulate ROS levels as well as serving as a source of much controversy.

## 5. Increasing ROS as an Anticancer Therapy

Recently, prooxidant cancer therapy has attracted much attention based on the rationale that ROS are responsible for triggering cell death and reversing chemoresistance in tumor cells. This rationale is based on the “ROS threshold concept” initially proposed by Kong et al. wherein normal cells and cancer cells were discriminated based on their differential susceptibility to combat against oxidative stress [64]. The threshold concept argues that cells adapt progressively to increasing concentrations of ROS by producing antioxidants, from an adaptive proliferation, passing through the equilibrium/balanced state, and finally, after the ROS level surpasses certain threshold levels, the cells are eliminated by apoptosis. Namely, treatment of cancer cells with ROS-inducing anticancer agents exceeds the threshold for ROS limits in cancer cells (which is much higher than that of normal cells), and this results in activation of cell death pathways (Figure 4). Indeed, it has been reported that piperlongumine, a natural product isolated from the plant species *Piper longum* L., can selectively kill cancer cells by increasing ROS levels without affecting normal tissues, including rapidly proliferating nontumor cells [67]. Thus, the induction of oxidative stress can lead to the preferential killing of cancer cells, and ROS-inducing drugs have now become a highly effective category of mechanism-based agents for individual and combined cancer chemotherapies (Table 1). In general, prooxidant cancer therapy could be performed by two means: (1) inducing ROS generation and (2) inhibiting the antioxidative defense systems in tumor cells (Table 1). Induction of ROS generation can be achieved by various chemotherapy agents and ionizing radiation, as well as drugs that induce ER stress and inhibitors of the ubiquitin-proteasome pathway [68]. Inhibition of antioxidative defense systems can be attained by drugs that affect GSH (glutathione) metabolism and thioredoxin metabolism as well as drugs that affect glucose metabolism.

One emerging trend for targeting the antioxidant capacity of tumor cells is the functional relevance of prooxidant therapy to cancer stem cells (CSCs). CSCs are characterized by their properties for self-renewal capacity and chemo-/radio resistance [69–72]. Importantly, CSCs have an enhanced capacity to initiate and sustain tumor growth, which is critical for the progression and recurrence of malignant tumors after chemotherapy or radiotherapy [73]. Given the above biological characteristics of CSCs, it is easily imaginable that this unique subpopulation may have a high antioxidant defense system, which might contribute to cancer stemness and drug resistance. CD44, previously known as an adhesion molecule, is expressed in CSCs of various types of cancers [74–76]. Intriguingly, the CD44v, a variant isoform of CD44, protects gastric cancer CSCs by interacting and stabilizing xCT, a glutamate-cystine transporter [77]. xCT facilitates cystine uptake thus increasing intracellular GSH synthesis, thereby enhancing ROS defense mechanism [78]. Human gastrointestinal cancer cells with a high level of CD44 expression showed an enhanced capacity for GSH synthesis and defense against ROS, which contributes to chemo-/radio resistance of CSCs [77]. Intriguingly, Ishimoto et al. demonstrated the ablation of CD44 signaling by the xCT inhibitor sulfasalazine which suppresses CD44-dependent cancer cell expansion *in vivo* [77]. It also induced activation of p38 MAPK, a downstream target of ROS, and expression of the gene for the cell cycle inhibitor p21^CIP1/WAF1^. This study highlighted the importance of CD44, in particular that of CD44v, in the protection of CSCs from high levels of ROS in the tumor microenvironment and further provides a rationale for CD44v-targeted therapy to impair ROS defense in cancer cells and sensitize them to currently available treatments.

## 6. Limitations and Possible Side Effects Associated with FTY720

As with all medications, FTY720 is not without risk of adverse event, and the most common adverse effect is being dose dependent [79]. Among a variety of side effects associated with FTY720, fatigue, nasopharyngitis, and influenza have been reported more frequent than others [80]. FTY720 has an immunosuppressive effect and thus increases the risk of serious infections. In fact, a recent routine EU review identified 54 reports of opportunistic systemic fungal infections, including 9 fatal cases of cryptococcal meningitis, over 397,764 patient years of exposure since marketing [81]. In addition to side effects common to immunomodulatory therapy, FTY720 was reported to cause cardiovascular complications, macular oedema, and brain inflammation [82]. These side effects are speculated as the result of interactions with more than one S1P receptor subtype [83].

Importantly, the anticancer property of FTY720 is largely independent of effects on S1P receptors [24]. Therefore, one way to minimize the side effects associated with FTY720 is to develop the analogues, like OSU-2S, that are devoid of immunosuppressive effect and interaction with S1P receptors. Reducing doses of FTY720 is another way to minimize the risk of adverse event although doses required for the anticancer effects (5 or 10 mg/kg/day) are higher than those used in multiple sclerosis models (<0.5 mg/kg/day) [4, 9, 13, 84]. Strategy to enhance the efficacy of FTY720 for anticancer therapy is needed.

## 7. Combination Therapy between FTY720 and Other ROS-Inducing Chemotherapies

Several combination therapies between FTY720 and various chemotherapy agents as well as molecular-targeted therapeutics have been performed. Of note, cisplatin, a well-established chemotherapy agent with ROS-inducing properties, was shown to induce enhanced cytotoxicity against human melanoma cell lines and ovarian cancer cell lines by combining with FTY720 [40, 85]. Notably, FTY720 and cisplatin synergistically induce the death of cisplatin-resistant melanoma cells through the downregulation of the PI3K pathway and the decrease in epidermal growth factor receptor expression (Table 2) [85]. In addition, FTY720 shows promising preclinical activity in mantle cell lymphoma (MCL) and sensitizes MCL cells to milatuzumab- (anti-CD74 humanized antibody-) mediated cell death [86]. Expectedly, the *in vitro* cytotoxicity of two novel bispecific anti-CD20-/CD74 antibodies to MCL lines was enhanced significantly by combining with FTY720.

**Figure 5:**
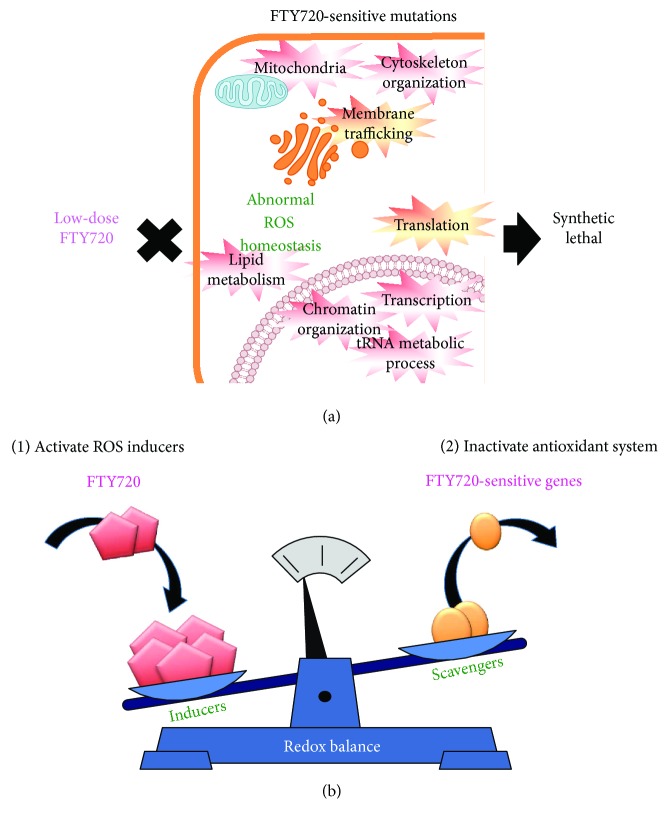
Strategies for combination therapy by manipulating ROS levels via FTY720-sensitive genes. (a) Functional categories of FTY720-sensitive genes and the concept of the synthetic lethality between mutations in FTY720-sensitive genes and FTY720 treatment. Mutation in the FTY720-sensitive genes induced elevated ROS levels. Combination of which is further increased upon FTY720 treatment. (b) Prooxidant therapy by combining (1) ROS generation by FTY720 and (2) antioxidant-inhibiting therapies by mutation in FTY720-sensitive genes.

As mentioned earlier, a chemical genetic approach using fission yeast has successfully reproduced ROS-mediated cell death phenotypes and resultant activation of the SAPK signaling pathway in mammals. Sugiura's group recently extended their research by pursuing a genome-wide screen for gene deletion mutants with enhanced cytotoxicity against FTY720 treatment [87]. Their aim is to investigate the ROS-related cytotoxic effects of FTY720 independently of its effects as a sphingosine-1-phosphate analogue on a genome-wide scale. They identified 49 FTY720-sensitive mutants which showed severe cell growth defects in the presence of a low-dose FTY720, wherein the wt cells can normally grow. Further characterization revealed that a strikingly high-number of these FTY720-sensitve mutants exhibited enhanced ROS accumulation in the absence of FTY720 treatment, indicating that these gene products are functionally involved in the ROS scavenging/defense system (Figure 5(a)). Notably, these FTY720-sensitve mutant cells exhibited markedly higher ROS levels upon FTY720 treatment as compared with the wt cells, thus suggesting that the excess ROS accumulation and an imbalance in ROS signaling play a key role as a key determinant of FTY720 toxicity (Figure 5(a)) [87]. The 49 gene products are functionally categorized in the biological processes involved in metabolic processes, transport, transcription, translation, chromatin organization, cytoskeleton organization, and intracellular signal transduction, thus revealing the presence of a complex regulatory network of FTY720-mediated ROS homeostasis, involving mitochondria, endosomes, transcription, translation, and tRNA and chromatin modifications (Figure 5(a)) [87].

Especially, several components and regulators of the Elongator complex have been identified of which deletion enhanced FTY720-induced cell death effect [87]. Elongator is an evolutionary highly conserved complex and has been reported to be a histone acetyltransferase complex involved in the elongation of RNA polymerase II transcription [88]. The molecular basis for the Elongator function in the FTY720-mediated ROS homeostasis would be that the lack of a functional Elongator complex resulted in oxidative stress phenotypes due to its contribution to tRNA modification and subsequent translation inefficiency of certain stress-induced mRNAs governed by SAPK and the downstream Atf1 transcription factor [89]. The SAPK signaling pathway and its downstream transcription factor Atf1 play critical roles in oxidative stress responses by regulating gene expression of various antioxidative enzymes, including catalase and superoxide dismutase. Therefore, FTY720-sensitive mutants involved in “gene expression processes” related to oxidative stress responses may affect the SAPK-dependent gene expression of antioxidative enzymes.

**Figure 6:**
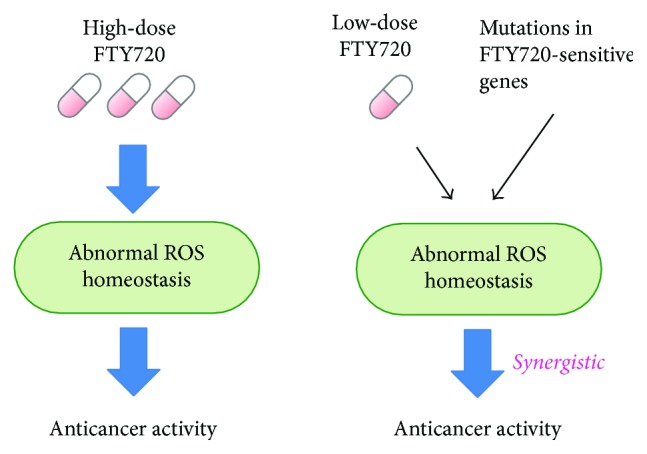
Possible clinical implications of mutations in FTY720-sensitive genes in combination with FTY720 for cancer therapy. In comparison with the use of FTY720 as a monotherapy, less dose of FTY720 is needed to induce apoptosis via ROS dysregulation in combination with therapies targeting FTY720-sensitive genes.

So, what is the clinical value of this FTY720-sensitive gene catalogue in terms of the efficacy of Fingolimod in cancer therapy? First, identification of new potential targets for combination cancer therapy as a means to enhance the antitumor efficacy of FTY720 was clearly shown by the list of FTY720-sensitive genes. As mentioned earlier, prooxidant cancer therapy could be performed by inducing ROS generation or by inhibiting the antioxidative defense systems. Importantly, deletion of the FTY720 sensitive genes per/se induced higher ROS levels and that FTY720 treatment further stimulated markedly enhanced ROS accumulation. From these observations, these gene products function in the ROS defense mechanism and combination of ROS induction by FTY720 (mechanism 1) with mutation in FTY720-sensitive genes (mechanism 2) induced higher ROS levels and cell death (Figure 5(b)). Therefore, as compared with FTY720 as a single regimen, the combination between mutation/inhibition of FTY720-sensitive genes and FTY720 treatment would require low-dose FTY720 to attain anticancer activity (Figure 6). This would be beneficial based on the finding that the doses required for the anticancer effects of FTY720 (5 or 10 mg/day) are higher than those used in multiple sclerosis models (<0.5 mg/day) in animal models [4, 9, 13, 84].

Therefore, the chemical genomics data using fission yeast not only help explain how cells fight the toxicity of FTY720 but also give us a sense of how we might be able to enhance the antitumor properties of FTY720.

## Figures and Tables

**Table 1:** Classification of anticancer treatments based on their role in ROS homeostasis.

	Name	Mechanism of action	Cancer types	Ref.
*Promotes ROS generation*				
Anticancer agent	Elesclomol	The induction of ROS and oxidative stress		[90]
Proteasome inhibitor	Bortezomib	Promotes ROS production via the endoplasmic reticulum system and apoptosis	Mantle cell lymphoma, nasopharyngeal carcinoma	[91, 92]
Polyphenolic compound	Curcumin	Promotes ROS generation through severe ER stress and growth inhibition/apoptosis	Metastatic colorectal cancer	[93]
Natural alkaloid	Cepharanthine (CEP)	Induces ROS production and promotes apoptosis through the mitochondrial signaling pathway	Choroidal melanoma	[94]
DNA intercalator	Adriamycin	Induces cell death that occurs probably due to a reduction in intracellular ROS formation, leading to induce p21 expression, a potent cyclin-dependent kinase inhibitor	Breast cancer	[95]
Platinum-based antineoplastic agent	Cisplatin	Induces a mitochondrial-dependent ROS response	Nonsmall lung cancer; the prostate cancer	[96]
A voltage-dependent anion channel (VDAC)-binding compound	Erastin	Induces ROS production and caspase-dependent apoptosis	Colorectal cancer	[97]
A constituent of many edible cruciferous vegetables including broccoli	Sulforaphane	Inhibits thyroid cancer cell proliferation, migration, and invasion and induces cell cycle arrest and apoptosis through a ROS-dependent pathway	Prostate cancer	[98]
An inhibitor of N-glycosylation	Tunicamycin	Induces ER stress and promotes ROS-mediated mitochondrial apoptosis by activating mTORC1 through the eNOS-RagC pathway	Prostate cancer	[99]
Hsp90 inhibitor	Geldanamycin	Increases intracellular calcium levels and ROS production and leads to ER stress-induced mitochondrial-mediated apoptosis	Brain tumor	[100]
*Inhibits the antioxidant system*				
Glutathione synthesis inhibitor	Buthionine sulfoximine (BSO)	Induces oxidative stress by inhibiting the activity of *γ*-glutamylcysteine synthetase, an enzyme in the GSH synthesis pathway	Melanoma; ovarian and breast cancer; chronic myeloid leukemia	[101, 102]
Inorganic compound	Arsenic trioxide	Induces growth inhibition and apoptosis through hydrogen peroxide generation, GSH depletion and Trx1 downregulation	Small cell lung cancer (SCLC)	[103]
Inhibitor of thioredoxin	PX-12	Increases the percentages of GSH-depleted cells and induces G2/M-phase arrest and Bax-mediated and ROS-dependent apoptosis	Lung cancer cells	[104]
A potent xCT inhibitor	Sulfasalazine	Increases the ROS accumulation and decreasing the GSH	Liver cancer	[105]

**Table 2:** Combination therapies with FTY720.

Combination of FTY720 with	Mechanism of action	Cancer types	Reference
Cisplatin	The downregulation of the PI3K/Akt/mTOR pathway and the decrease in EGFR expression	Human melanoma	[85]
Cisplatin	Autophagy	Ovarian cancer	[106]
Doxorubicin and etoposide	The promotion of apoptosis and the inhibition of P-glycoprotein and multidrug-resistance protein 1	Colon cancer	[107]
The fully humanized monoclonal antibody milatuzumab	The disruption of the autophagic-lysosomal pathway ROS?	Mantle cell lymphoma	[86]
Temozolomide	Apoptosis	Brain tumor	[5]
5-Fluorouracil, SN-38, and oxaliplatin	PP2A activation and apoptosis	Colorectal cancer human colorectal cancer	[108]
Rapamycin	Autophagy, apoptosis, and necrosis induction in ROS-JNK-p53 loop-mediated PI3K/AKT/mTOR/p70S6K-dependent manner	Pancreatic cell	[109]
Sorafenib	Cell cycle arrest and apoptosis, possibly through blockage of autophagy	Hepatocellular carcinoma	[110]
Gemcitabine	The inhibition of the S1P signaling pathway and both HIF1*α* and HIF2αaccumulation	Clear cell renal cell carcinoma	[111]
